# Association atrésie de l'oesophage type 3 - microcéphalie: Un syndrome de Feingold incomplet?

**Published:** 2012-12-20

**Authors:** Mick Yapongombo Shongo, Toni Kasole Lubala, Sébastien Musanzayi Mbuyi, Paul Ilunga Makinko, Dieudonné Tshikwej Ngwej, Felix Numbi Kabange

**Affiliations:** 1 Faculté de Médecine, Université de Lubumbashi, BP1825, Lubumbashi, République Démocratique du Congo; 2 Centre Interdisciplinaire de Génétique au Congo, CIGEC, Lubumbashi, République Démocratique du Congo

**Keywords:** Atrésie œsophagienne, microcéphalie, Feingold, esophageal atresia, microcephaly, Feingold

## Abstract

L'atrésie de l'oesophage est une des malformations digestive les plus fréquentes en néonatologie. Il existe 5 types anatomiques, selon la présence et le siège de la fistule oeso-trachéale. Le diagnostic anténatal est difficile. A la naissance, le diagnostic est suspecté en salle de travail, devant l’échec du passage systématique d'une sonde digestive jusque dans l'estomac. Des trouble de la déglutition, une hyper sialorrhée, une détresse respiratoire sont ensuite retrouvés. Une fistule oeso-trachéale peut être isolée et suspectée devant des épisodes de cyanose et de dyspnée lors des tétées ou en cas de survenue d'une pneumopathie d'inhalation. Dans notre cas, nous avons observé une microcéphalie primaire. Une association microcéphalie et malformation digestive et des membres a été décrite sous le nom de syndrome de Feingold. Chez notre patient, aucune malformation des membres n'a été observée.

## Introduction

L'atrésie de l'oesophage est une des malformations digestive la plus fréquente en néonatologie. Elle survient approximativement chez 1 nouveau ne sur 3500 naissances vivantes [1]. Il existe 5 types anatomiques, selon la présence et le siège de la fistule oeso-trachéale. Le diagnostic anténatal est difficile. À la naissance, le diagnostic est suspecté en salle de travail, devant l’échec du passage systématique d'une sonde digestive jusque dans l'estomac. Des troubles de déglutition, une hyper sialorrhée, une détresse respiratoire sont ensuite retrouvés. Une fistule eoso-trachéale peut être isolée, et suspectée devant des épisodes de cyanose et de dyspnée lors des tétées, une pneumopathie d'inhalation. Une association microcéphalie et malformation digestive et des membres a été décrite sous le nom de syndrome de Feingold [2].

Cet article présente le cas d'un nouveau-né reçu au service de néonatologie des cliniques universitaires de Lubumbashi transféré d'un centre de santé périphérique durant les 24 premières heures de vie pour prise en charge d'une détresse respiratoire.

## Patient et observation

Nous décrivons un nouveau-né de 6 heures de vie, de sexe masculin, ayant présenté pour mensuration: poids 2650get périmètre crânien 30 cm. Il est né à terme, cadet d'une fratrie de 4. Les parents n'ont pas rapporté d'antécédent de malformations dans la famille. La grossesse a été marquée par un hydramnios découvert au 2ème trimestre. Les autres éléments ne sont pas contributifs. Il n'y avait pas de dysmorphie faciale et aucune autre malformation externe visible.

Le patient étaiten détresse respiratoire modérée (cotation de Sylverman à 4), tachypnéique (FR à 55 par minute), tachycarde (FC à 110 par minute) et présentait des secrétions buccales ainsi que des râles crépitant aux poumons. L'abdomen était ballonné, luisant avec présence d'une circulation collatérale visible. Il était par ailleurs flatulent, peu dépressible et des bruits péristaltiques étaient perçus. Chaque tentative d'alimentation se solde par une écume à la bouche. Nous avons conclu en un nouveau-né à terme avec suspicion d'atrésie de l'oesophage et iléus méconial probable.

**Figure 1:**
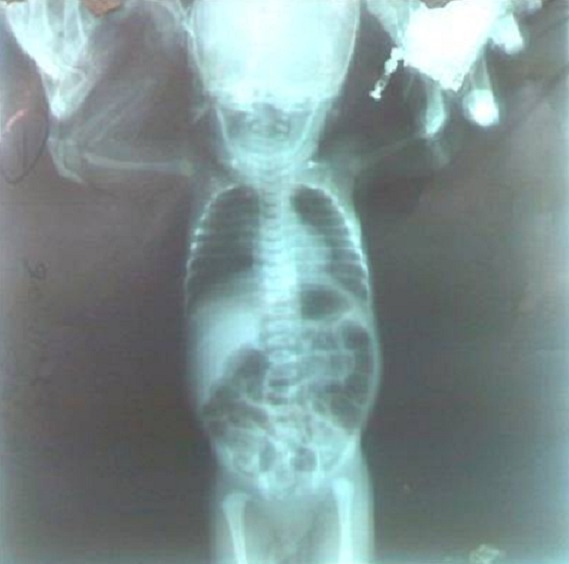
Radiographie thoraco-abdominale sans préparation réalisée au 2ième jour a révélé un enroulement de la sonde au niveau de la 4ième vertèbre cervicale et de nombreuxgaz au niveau des intestins

**Figure 2:**
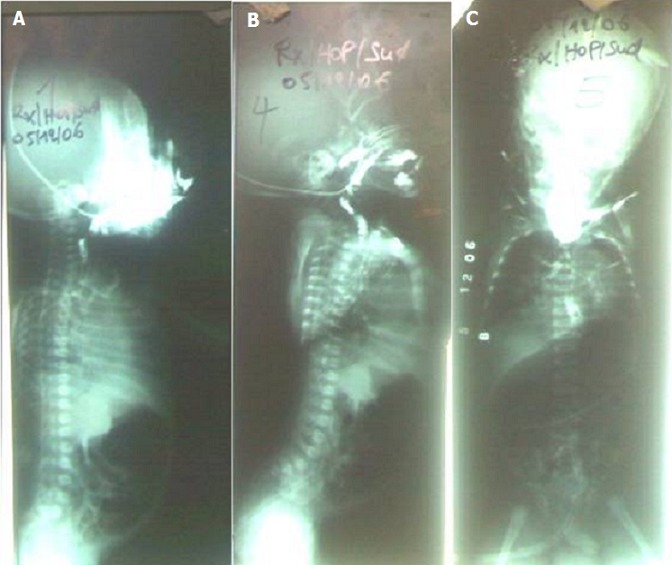
A) Le transit œsogastroduodénal à la Gastrografine a montré une destruction totale de l’œsophage au niveau de la jonction du tiers proximal et tiers moyen; B) Le cliché montre l'opacification de la trachée et des bronches souches, en plus l'existence d'une communication entre la trachée et le tiers moyens de l’œ'oesophage; C) Le début d'inondation des poumons par le produit de contraste

Son évolution était marquée par: au 4ème jour une émission abondante de méconium et apparition de la fièvre; au 11ème jour s'ajoute une conjonctivite; L’échographie abdominale était normale. La Radiographie abdominale sans préparation réalisée au 2ième jour a révélé un enroulement de la sonde au niveau de la 4ième vertèbre cervicale et de nombreux gaz au niveau des intestins (Figure 1). Le transit oesogastroduodénal à la Gastrografinea montré une destruction totale de l'eosophage au niveau de la jonction du tiers proximal et tiers moyen (Figure 2), après reflux et inhalation du produit de contraste le cliché montre l'opacification de la trachée et des bronches souches, en plus l'existence d'une communication entre la trachée et le tiers moyens de l'oesophage (Figure 2). L'inondation des poumons n'a pas permis de poursuivre l'examen (Figure 2).

Au total, le tableau était compatible avec une atrésie de l'oesophage type 3 syndromique, puisqu'associée à une microcéphalie. Le décès est survenu au 13ème jour de vie avant qu'une prise en charge chirurgicale ne soit initiée.

## Discussion

Dans notre cas il sagissait dune atrésie au niveau de la 4ième vertèbre thoracique et fistule entre le segment inferieur et la trachée. Cest la variété la plus commune [3]. La classification originale est l'oeuvre de Vogt en 1929 [3]. Ladd en 1944 puis Gross en 1953 modifie cette classification mais c'est Kluth en 1976 qui décrit 10 types majeurs [4, 5]. Le diagnostic d'une atrésie de l'oesophage peut déjà être suspecté à la période prénatale par un hydramnios associé à l'absence ou la présence d'un petit estomac visible à l’échographie dès la huitième semaine de gestation [6]. Dans notre cas, un hydramnios a été observé au 2ème trimestre de grossesse et l’échographie abdominale était normale. La présence de gaz dans l'estomac et les intestins signe une fistule distale trachéo oesophagienne. C'est exactement ce que nous avons observé dans notre cas.

Le nouveau-né ne peut s′alimenter; la salive et le lait ne pouvant descendre dans l′estomac va refluer dans l′oesophage et passer par fausse-route dans les voies respiratoires justifiant les secrétions buccales observées et la dyspnée. Le liquide gastrique remonte directement dans les poumons par la fistule trachéo oesophagienne. Il est conseillé fortement de rechercher d'autres anomalies associées [1, 7]. Dans notre cas, nous avons observé une microcéphalie primaire. Une association microcéphalie et malformation digestive et des membres a été décrite sous le nom de syndrome de Feingold [2]. Dans notre cas aucune malformation des membres n'a été observée. Un petit nombre de ces malformations sont associés aux anomalies chromosomiques [1]. Les cas familiaux d'atrésie oesophagienne sont extrêmement rares. L′atrésie de l'oesophage est 2 à 3 fois plus commune chez les jumeaux. Le risque global de répétition d′atrésie oesophagienne chez les enfants de mêmes parents est d'environ 1% [8]. Dans notre cas il est unique sur 877 nouveaux nés vivants aux Cliniques Universitaires de Lubumbashi au cours de l'année 2006. La chirurgie représente le seul recours possible [9].

## Conclusion

Malformation incompatible avec la vie, l′atrésie de l′oesophage est le type même d′une malformation locorégionale. Une association microcéphalie et malformation digestive et des membres a été décrite sous le nom de syndrome de Feingold. Dans notre cas cette atrésie était associée à une microcéphalie sans qu'aucune malformation des membres ne soit observée. Il s'agissait probablement d'une forme incomplète du syndrome de Feingold.
